# Quality of Life, Depression, and Psychological Distress Among Patients With Rheumatoid Arthritis Treated With Biologics

**DOI:** 10.7759/cureus.72384

**Published:** 2024-10-25

**Authors:** Diellor Rizaj, Artidon Kelmendi

**Affiliations:** 1 Medical Sciences, University for Business and Technology (UBT), Prishtina, ALB; 2 Rheumatology, University Clinical Center of Kosovo, Prishtina, ALB

**Keywords:** biologics, depression, psychological distress, quality of life, rheumatoid arthritis

## Abstract

The introduction of biologics has improved the management of rheumatoid arthritis (RA), enhancing disease control and quality of life for patients. However, despite these advancements, some individuals still experience distress and depression due to the effects of treatment and concerns about the outcomes of biologic therapy. This study aimed to evaluate the current quality of life, level of distress, and depression among RA patients treated with biologics, which could serve as a basis for intervention. The Quality of Life in Rheumatoid Arthritis Instrument II (QoL-RAII), Kessler Psychological Distress Scale (KPDS), and Beck Depression Inventory (BDI) were used to gather the necessary data. These standardized questionnaires were randomly administered to 100 RA patients undergoing biologic treatment. The data were then tabulated and analyzed using the mean and Pearson r correlation.

The study's results revealed a low average quality of life and a very high level of psychological distress among the respondents. Additionally, there was an extreme level of depression observed in RA patients. Furthermore, a significant correlation was found between quality of life and the level of psychological distress among the patients. The findings concluded that RA patients have poor quality of life, experience very high levels of psychological distress, and suffer from extreme depression. The study underscored the urgent need for mental health professionals to provide immediate intervention.

## Introduction

Mental health is as important as physical health. According to the World Health Organization, mental health is a state of well-being in which individuals realize their abilities, cope with the normal stresses of life, work productively and fruitfully, and contribute to their community [1]. Physical diseases impede individuals from living fulfilling lives, as prolonged pain can lead to distress and depression [2].

The diseases associated with depression include diabetes mellitus, asthma, cancer, and rheumatoid arthritis (RA). Among these, arthritis, particularly rheumatoid arthritis, affects about 1% of the adult population, which represents a low worldwide prevalence [3]. However, Sturgeon et al. noted that depression, distress, and anxiety are strongly linked to RA due to its biologic mechanisms and the psychological consequences of treatment [4]. The mental health of RA patients is not the only aspect at risk; their quality of life is also impaired. The physical sensations experienced by RA patients, such as fatigue, pain, and stiffness, further affect their quality of life [5].

Quality of life refers to the ability of patients to enjoy everyday activities. It encompasses not just one aspect but various areas of life, including social, emotional, and physical dimensions. The quality of life for patients suffering from rheumatoid arthritis declines in terms of their autonomy, physical health, and environment [6]. Biologic medicines are the standard treatment for RA. In fact, a study by Boyadzieva et al. revealed that RA patients who received biologics experienced significant improvements in their quality of life [7]. Although treatment enhances the quality of life for patients, continuous treatment is often hindered by the high costs associated with biologic medicines. Additionally, factors such as age, distress, and depression, commonly experienced by RA patients, can have negative consequences on their quality of life [8,9].

According to the American Psychological Association, depression is a mood disorder that causes persistent feelings of hopelessness, loneliness, anhedonia, and low interest. Various studies have reported that depression is associated with a lower quality of life [10]. For example, a study by Tanski et al. among samples of rheumatoid arthritis (RA) patients revealed that a lower quality of life among RA patients is strongly correlated with depression [9]. In addition to age, depression, and physical symptoms, distress also influences the quality of life of RA patients [11]. The recurring experience of pain causes RA patients to gradually suffer from psychological distress, particularly in the early stages of the disease [12]. The burden of pain increases the susceptibility of RA patients to distress, anxiety, and depression [13].

Our study aimed to evaluate the relationship between the quality of life among RA patients and their levels of psychological distress and depression.

## Materials and methods

The study utilized a quantitative research design, specifically employing a descriptive method to statistically describe the condition of RA patients in terms of their quality of life, depression, and psychological distress. The descriptive method involves collecting data to test hypotheses or answer questions about the current status of the subjects in the study. Additionally, a correlational design was used to achieve the aim of this study, as it determined the correlation between variables. This was instrumental in identifying the significance of the relationships between quality of life, depression, and psychological distress.

The research was conducted through face-to-face data collection at the University Clinical Center of Kosovo Rheumatology Clinic. Professionally translated surveys were used to gather data. The participants were 100 RA patients from the rheumatology clinic, randomly selected for this study. Simple random sampling was utilized, and data were gathered from January 2022 to June 2023. The data collection tools were administered using printed forms. The questionnaires included information about the respondents' demographic profiles and research-related questions. The respondents were given sufficient time to complete the questionnaires, and clarifications regarding the content were provided when needed. Once all questionnaires were completed and collected, they were securely stored. The Scientific Ethics Committee of the University Clinical Center of Kosovo approved the study (approval number: 11490).

From the gathered results, scores from the surveys were tabulated, and the data were further processed and analyzed. Moreover, we ensured compliance with the Data Privacy Act during the data collection process. Thus, the study involved individuals who demonstrated moral and ethical characteristics. Consent forms were secured prior to the main data collection, and all personal information was kept confidential. Participants were informed of their right to withdraw at any time and to refuse participation in achieving the study’s objectives if it went against their will.

To qualify for the study, the following inclusion criteria had to be met: participants, male or female, diagnosed with RA, aged 18 years or older, and currently receiving biologic treatment. The exclusion criteria for RA patients were set to minimize the risk of false results, excluding those with other neurodegenerative disorders. The approved and signed cover letter, along with detailed instructions, was attached. This procedure allowed the respondents to clearly understand the purpose of the study and provide informed consent, thereby facilitating their participation.

Three data-gathering tools were used in the study, namely the Beck Depression Inventory (BDI) by Beck et al., the Kessler Psychological Distress Scale (KPDS) by Kessler et al., and the Quality of Life in Rheumatoid Arthritis Instrument II (QoL-RAII) by Isnardi et al. [14-16]. The first tool demonstrated high internal consistency, with a Cronbach’s alpha of 0.96, and good construct validity. These psychometric properties correlate well with those of the EQ-5D-3L, which has an r = 0.6. The BDI showed a high test-retest reliability of 0.93, while the KPDS had a reliability of 0.74. Furthermore, scores on the QoL-RAII were averaged, with higher scores indicating a higher quality of life, while BDI scores were summed. The BDI scores were interpreted according to the levels specified in the scale’s manual: 1-10 as normal, 11-16 as mild, 17-20 as borderline, 21-30 as moderate, 31-40 as severe, and 40 and above as extreme. Similarly, KPDS scores were added and categorized as follows: 1-19 as low, 20-24 as moderate, 25-29 as high, and 30-50 as very high.

SPSS Statistics version 26 (IBM Corp. Released 2019. IBM SPSS Statistics for Windows, Version 26.0. Armonk, NY: IBM Corp.) was used for all data computations in this study, as it is the most suitable program for both qualitative and quantitative data analysis in the social sciences. The gathered data were interpreted using the following statistical tools: first, the mean of the raw scores was used to assess the quality of life and the level of psychological distress and depression of the patients, as outlined in the manuals of the adopted tools. Finally, partial correlation analysis was employed to test the associations between quality of life and the levels of psychological distress and depression among RA patients treated with biologics.

## Results

**Figure 1:**
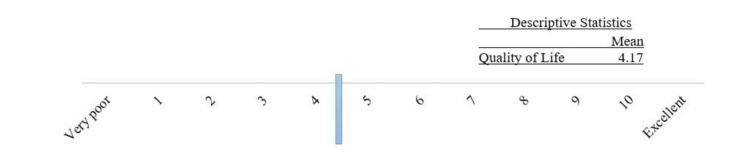
Quality of life among RA patients treated with biologics RA: rheumatoid arthritis

The quality of life of the respondents was measured using the QoL-RAII, a horizontal scale ranging from 1 to 10, where 1 indicates very poor and 10 indicates excellent. The mean score of the patients was 4.17, indicating that their quality of life was between 4 and 5. Scores ranged from 4 points (closer to very poor) to 6 points (approaching excellent). As shown in Figure 1, their quality of life is less than half of the scale for very poor quality of life. Further analysis revealed that patients with RA rated their quality of life poorly, considering factors such as physical ability, pain, exhaustion, health, mood, support, and interactions with family and friends.

**Figure 2:**
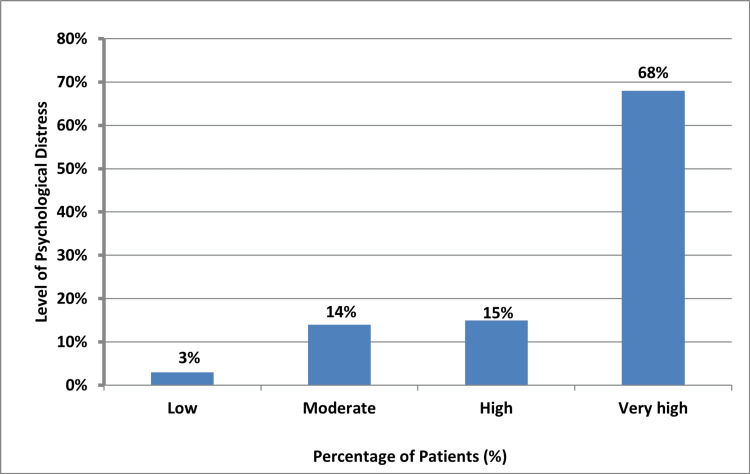
Percentage distribution of the level of psychological distress in RA patients treated with biologics RA: rheumatoid arthritis

The percentage distribution in Figure 2 shows that 3% of the respondents experienced a low level of psychological distress, 14% experienced moderate distress, 15% experienced a high level of psychological distress, and 68% exhibited a very high level of psychological distress.

The general level of psychological distress among RA patients is presented in Table 1. Mean scores were computed to determine the level of distress for the overall representative sample, as instructed in the Kessler manual. The mean score is 32.27, which is interpreted as a very high level, as indicated in the scoring and interpretation of the scale.

**Table 1:** Overall level of psychological distress in RA patients SD: standard deviation, RA: rheumatoid arthritis

Descriptive statistics	Level of psychological distress
Mean ± SD	32.87

**Figure 3:**
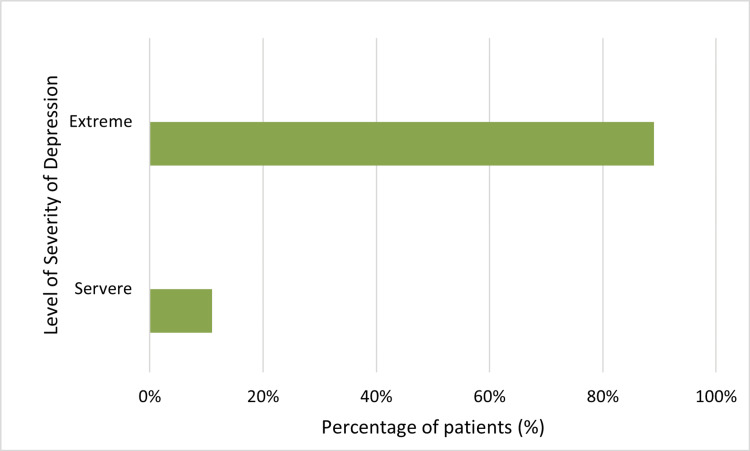
Percentage distribution of depression severity in RA patients RA: rheumatoid arthritis

The percentage distribution of the level of depression among the respondents is shown in Figure 3. Eighty-nine percent of the respondents exhibited an extreme level of depression, while eleven percent experienced severe depression. Nevertheless, the majority of RA patients treated with biologics experience extreme depression.

The overall level of depression is presented in Table 2. The mean score was examined to determine the level of depression among the respondents. The computed mean is 46.27, which is interpreted by the BDI Manual as extreme. The findings revealed that the level of depression among the respondents was indeed at an extreme level, implying the need for additional antidepressant therapy or psychotherapy interventions.

**Table 2:** Overall depression level SD: standard deviation

Descriptive statistics	Level of depression
Mean ± SD	46.27

Table 3 shows the correlation test using partial correlation between quality of life and the respondents’ levels of psychological distress and depression. There was a significant relationship between quality of life and the level of psychological distress (r=-0.260, p=0.0009). This indicates that the quality of life of RA patients significantly affects the level of psychological distress they experience. However, there was no significant relationship between quality of life and the level of depression (r=0.086, p=0.394).

**Table 3:** Correlation between quality of life, psychological distress, and depression among RA patients treated with biologics ** correlation is significant at the 0.05 level (two-tailed)

		Correlation	
	Quality of life	Level of psychological distress	Level of depression
Pearson r	1	-0.260^**^	0.086
Sig.		0.009	0.394

As shown in Table 3, the results revealed a significant negative correlation between quality of life and the level of psychological distress, implying that the better the quality of life, the lower the level of psychological distress.

## Discussion

The study aimed to assess the quality of life, level of psychological distress, and depression among RA patients treated with biologics and to determine the relationship between their quality of life and their levels of psychological distress and depression. According to the results, RA patients demonstrated below-average quality of life, indicating that their quality of life was not acceptable. This is quite surprising since patients are undergoing biologic treatment; however, previous studies have reported various underlying factors that can possibly affect the quality of life of patients, such as age, sleep duration, lifestyle habits, employment, and economic conditions [6,17,18]. This may be connected to the level of psychological distress experienced by RA patients.

The results revealed a relationship between quality of life and psychological distress among patients. The fact that patients rated their lives as poor might have been influenced by the level of psychological distress they experienced. A limitation of this study is whether patients rated their quality of life as poor because it truly was poor or because they poorly rated it as a result of the distress they were experiencing. This can be confirmed after an intervention is implemented to address the high psychological distress among RA patients. Despite the clinic's efforts to provide treatment to RA patients and help them overcome the consequences of the disease to live fulfilling lives, patients still rate their quality of life as poor. This contradicts the findings of Tanski et al., who reported an average quality of life among RA patients treated with biologics [9].

Furthermore, the psychological distress of RA patients, based on the results of the KPDS, was very high. It is expected, based on previous studies, that patients treated with biologics often experience renewed mobility and enhanced hope [19]. However, in this study, the psychological distress of the patients was extreme. The dramatic changes that patients must undergo to comply with treatment may be distressing for both patients and their families; having this mental condition while undergoing treatment may lead to patients being less responsive to treatment [3]. This finding contradicts the conclusions of Silke et al., who reported that patients with RA often struggle with distress, but that it is disease-specific rather than psychological [11].

Regarding the level of depression, the present study indicated that the patients' overall level of depression was extreme, suggesting that RA patients treated with biologics are grappling with significant levels of depression. This finding correlates with the results of Baghdadi et al., who reported that higher depression scores are found in RA patients treated with biologics, particularly disease-modifying antirheumatic drugs [20]. Another study by Bournia et al. showed that the level of depression among RA patients treated with biologics is influenced by switching to another biologic treatment [21].

Moreover, the results showed that the relationships between quality of life and psychological distress among RA patients were significant, indicating that the variables are related to each other. Thus, the quality of life of patients contributes to their level of psychological distress. Furthermore, quality of life and the level of depression were not significantly correlated, suggesting that the extreme level of depression experienced by RA patients is not related to their quality of life. This finding contradicts that of Tanski et al., who reported a significant negative correlation between quality of life and depression among RA patients treated with biologics [9].

Limitations

The limitation of our study was the small number of subjects. Furthermore, the study group was enrolled in one clinic center and was limited to patients treated with biologics. In the future, the study sample should be larger, and a cohort study should be conducted to compare the outcomes in a patient group before and after receiving biologic treatment. Another limitation of the study was that the patients included differed regarding the length of disease and the treatment they received. It would also be beneficial to follow up with patients from the onset of symptoms and throughout the follow-up of therapy or disease progression to evaluate quality of life, psychological distress, and depression. Finally, even in our study, standardized questionnaires pertaining to quality of life, depression, and psychological distress assessments make it difficult to compare the results obtained with findings in other studies that used different standardized tools for quality of life, depression, and psychological distress assessments.

## Conclusions

RA patients rated their quality of life as poor, considering the pain, mood, exhaustion, and support they received from family and friends. Therefore, there should be an environment where they can be taught how to cope with the various determinants affecting their quality of life. Furthermore, this study revealed that RA patients undergoing biologic treatment are experiencing extreme distress and depression. Thus, interventions and assistance from hospitals and families must be provided to address their mental health conditions. The well-being of patients must be secured and sustained to prevent the probability of ongoing extreme psychological distress and depression, which could hinder their compliance and response to ongoing treatment.

This study also revealed that the quality of life of RA patients negatively influences their level of distress. As the quality of life of patients decreases, their distress increases. Therefore, the quality of life of RA patients must be improved to reduce the distress they experience. We concluded in this study that the level of depression in RA patients is not influenced by quality of life. Hence, the level of depression among patients remains poor regardless of whether their quality of life is poor or excellent. Therefore, there must be a mental health intervention that focuses on managing the extreme depression experienced by patients.

## Appendices

Beck Depression Inventory

**Table 4:** Beck Depression Inventory Interpreting the Beck Depression Inventory (total score: levels of depression) 1-10: these ups and downs are considered normal, 11-16: mild mood disturbance, 17-20: borderline clinical depression, 21-30: moderate depression, 31-40: severe depression, over 40: extreme depression [14]

1.		
0		I do not feel sad.
1		I feel sad.
2		I am sad all the time and I can't snap out of it.
3		I am so sad and unhappy that I can't stand it.
2.		
0		I am not particularly discouraged about the future.
1		I feel discouraged about the future.
2		I feel I have nothing to look forward to.
3		I feel the future is hopeless and that things cannot improve.
3.		
0		I do not feel like a failure.
1		I feel I have failed more than the average person.
2		As I look back on my life, all I can see is a lot of failures.
3		I feel I am a complete failure as a person.
4.		
0		I get as much satisfaction out of things as I used to.
1		I don't enjoy things the way I used to.
2		I don't get real satisfaction out of anything anymore.
3		I am dissatisfied or bored with everything.
5.		
0		I don't feel particularl guilty.
1		I feel guilty a good part of the time.
2		I feel quite guilty most of the time.
3		I feel guilty all of the time.
6.		
0		I don't feel I am being punished.
1		I feel I may be punished.
2		I expect to be punished.
3		I feel I am being punished.
7.		
0		I don't feel disappointed in myself.
1		I am disappointed in myself.
2		I am disgusted with myself.
3		I hate myself.
8.		
0		I don't feel I am any worse than anybody else.
1		I am critical of myself for my weaknesses or mistakes.
2		I blame myself all the time for my faults.
3		I blame myself for everything bad that happens.
9.		
0		I don't have any thoughts of killing myself.
1		I have thoughts of killing myself, but I would not carry them out.
2	I would like to kill myself.	
3	I would kill myself if I had the chance.	
10.		
0	I don't cry any more than usual.	
1	I cry more now than I used to.	
2	I cry all the time now.	
3	I used to be able to cry, but now I can't cry even though I want to.	
11.		
0	I am no more irritated by things than I ever was.	
1	I am slightly more irritated now than usual.	
2	I am quite annoyed or irritated a good deal of the time.	
3	I feel irritated all the time.	
12.		
0	I have not lost interest in other people.	
1	I am less interested in other people than I used to be.	
2	I have lost most of my interest in other people.	
3	I have lost all of my interest in other people.	
13.		
0	I make decisions about as well as I ever could.	
1	I put off making decisions more than I used to.	
2	I have greater difficulty in making decisions more than I used to.	
3	I can't make decisions at all anymore.	
14.		
0	I don't feel that I look any worse than I used to.	
1	I am worried that I am looking old or unattractive.	
2	I feel there are permanent changes in my appearance that make me look unattractive.	
3	I believe that I look ugly.	
15.		
0	I can work about as well as before.	
1	It takes an extra effort to get started at doing something.	
2	I have to push myself very hard to do anything.	
3	I can't do any work at all.	
16.		
0	I can sleep as well as usual.	
1	I don't sleep as well as I used to.	
2	I wake up 1-2 hours earlier than usual and find it hard to get back to sleep.	
3	I wake up several hours earlier than I used to and cannot get back to sleep.	
17.		
0	I don't get more tired than usual.	
1	I get tired more easily than I used to.	
2	I get tired from doing almost anything.	
3	I am too tired to do anything.	
18.		
0	My appetite is no worse than usual.	
1	My appetite is not as good as it used to be.	
2	My appetite is much worse now.	
3	I have no appetite at all anymore.	
19.		
0	I haven't lost much weight, if any, lately.	
1	I have lost more than five pounds.	
2	I have lost more than ten pounds.	
3	I have lost more than fifteen pounds.	
20.		
0	I am no more worried about my health than usual.	
1	I am worried about physical problems like aches, pains, upset stomach, or constipation.	
2	I am very worried about physical problems and it's hard to think of much else.	
3	I am so worried about my physical problems that I cannot think of anything else.	
21.		
0	I have not noticed any recent change in my interest in sex.	
1	I am less interested in sex than I used to be.	
2	I have almost no interest in sex.	
3	I have lost interest in sex completely.	

Kessler Psychological Distress Scale

The Kessler Psychological Distress Scale [15] is a simple measure of psychological distress. The scale consists of 10 questions about emotional states, each accompanied by a five-level response scale. This measure can serve as a brief screening tool to identify levels of distress. It can be given to patients to complete, or alternatively, the questions can be read to the patient by the practitioner. In the context of injury management, the measure can be provided to patients when recovery is not proceeding as anticipated (for instance, between weeks four and six) and may highlight the need for more regular reviews or referrals to a specialist health provider, such as a psychologist.

Questions three and six do not need to be asked if the response to the preceding question was "none of the time." In such cases, questions three and six should automatically receive a score of one.

Scoring Instructions

Each item is scored from one ("none of the time") to five ("all of the time"). The scores of the 10 items are then summed, yielding a minimum score of 10 and a maximum score of 50. Low scores indicate low levels of psychological distress, while high scores indicate high levels of psychological distress.

Interpretation of Scores

The likelihood of having a mental disorder, indicated by psychological distress, is categorized as follows: scores of 10 to 19 suggest a likelihood of being well, scores of 20 to 24 indicate a likelihood of having a mild disorder, scores of 25 to 29 suggest a likelihood of having a moderate disorder, and scores of 30 to 50 indicate a likelihood of having a severe disorder.

**Table 5:** Kessler Psychological Distress Scale [15]

Please tick the answer that is correct for you:	All of the time (score 5)	Most of the time (score 4)	Some of the time (score 3)	A little of the time (score 2)	None of the time (score 1)
1. In the past 4 weeks, about how often did you feel tired out for no good reason?					
2. In the past 4 weeks, about how often did you feel nervous?					
3. In the past 4 weeks, about how often did you feel so nervous that nothing could calm you down?					
4. In the past 4 weeks, about how often did you feel hopeless?					
5. In the past 4 weeks, about how often did you feel restless or fidgety?					
6. In the past 4 weeks, about how often did you feel so restless you could not sit still?					
7. In the past 4 weeks, about how often did you feel depressed?					
8. In the past 4 weeks, about how often did you feel that everything was an effort?					
9. In the past 4 weeks, about how often did you feel so sad that nothing could cheer you up?					
10. In the past 4 weeks, about how often did you feel worthless?					

Quality of Life in Rheumatoid Arthritis Instrument II

**Table 6:** Quality of Life in Rheumatoid Arthritis Instrument II [16]

1. Considering your physical ability, how would you rate your quality of life?
Very poor 1 2 3 4 5 6 7 8 9 10 Excellent
2. Considering the help that you receive from family and friends, how would you rate your quality of life?
Very poor 1 2 3 4 5 6 7 8 9 10 Excellent
3. Considering the pain in your joints, how would you rate your quality of life?
Very poor 1 2 3 4 5 6 7 8 9 10 Excellent
4. Considering your level of tension, how would you rate your quality of life?
Very poor 1 2 3 4 5 6 7 8 9 10 Excellent
5. Considering only your health, how would you rate your quality of life?
Very poor 1 2 3 4 5 6 7 8 9 10 Excellent
6. Considering your fatigue/exhaustion, how would you rate your quality of life?
Very poor 1 2 3 4 5 6 7 8 9 10 Excellent
7. Considering your level of interaction with your family and friends, how would you rate your quality of life?
Very poor 1 2 3 4 5 6 7 8 9 10 Excellent
8. Considering your mood, how would you rate your quality of life?
Very poor 1 2 3 4 5 6 7 8 9 10 Excellent
